# LEISURE ACTIVITIES, MARITAL STATUS, AND COGNITIVE FUNCTIONING IN LATER LIFE IN MEXICO

**DOI:** 10.1093/geroni/igad104.0901

**Published:** 2023-12-21

**Authors:** Maria Monserud

**Affiliations:** University of Houston, Houston, Texas, United States

## Abstract

Prior research indicates that leisure activities can shape cognition in later life. However, little is known about whether the implications of specific group and individual leisure activities for older adults’ cognitive functioning can be contingent on marital status and gender and whether physical and mental health can make a difference in the associations between leisure participation and cognition. Moreover, research on the role of these factors in older adults’ cognitive functioning in Mexico has been scarce. Using data from the most recent wave (2018) of the Mexican Health and Aging Study, a nationally representative sample of Mexicans aged 50+, this study examines the implications of group and individual leisure activities for cognition among married and unmarried older men and women in Mexico while considering their physical health (i.e., vision, hearing, chronic conditions, and ADL and IADL limitations) and depressive symptoms. The results suggest that not only group-based but also individual activities can be predictive of cognitive functioning among older Mexicans. The findings also reveal some marital status and gender differences and similarities in the effect of certain leisure activities on cognition. Additionally, the results demonstrate that experiencing physical health challenges and depressive symptoms can prevent older Mexicans from deriving cognitive benefits from some leisure activities. Yet, despite poorer physical and mental health, certain leisure activities can still lead to better cognitive functioning in this population group. The insights from this study can be useful for the development of preventive strategies and intervention programs to improve cognitive health in later life.

